# SleepMFormer: An Efficient Attention Framework with Contrastive Learning for Single-Channel EEG Sleep Staging

**DOI:** 10.3390/brainsci16010095

**Published:** 2026-01-16

**Authors:** Mingjie Li, Jie Xia, Jiadong Pan, Sha Zhao, Xiaoying Zhang, Hao Jin, Shurong Dong

**Affiliations:** 1 State Key Laboratory of Brain-Machine Intelligence, Zhejiang University, Hangzhou 311121, China; 22331067@zju.edu.cn (M.L.); jiexia@zju.edu.cn (J.X.); 12431073@zju.edu.cn (J.P.); szhao@zju.edu.cn (S.Z.); 2 College of Information Science & Electronic Engineering, Zhejiang University, Hangzhou 310058, China; hjin@zju.edu.cn; 3 College of Computer Science and Technology, Zhejiang University, Hangzhou 310013, China; 4 Department of Neurology, Affiliated Mental Health Center, Hangzhou Seventh People’s Hospital, Zhejiang University School of Medicine, Hangzhou 310012, China; xyzhang@hz7hospital.com

**Keywords:** sleep staging, electroencephalography, sparse attention, supervised contrastive learning, efficient transformer, computational efficiency

## Abstract

Background/Objectives: Sleep stage classification is crucial for assessing sleep quality and diagnosing related disorders. Electroencephalography (EEG) is currently recognized as a primary method for sleep stage classification. High-performance automatic sleep staging methods based on EEG leverage the powerful contextual modeling capabilities of Transformer Encoder architectures. However, the global self-attention mechanism in Transformers incurs significant computational overhead, substantially hindering the training and inference efficiency of automatic sleep staging algorithms. Methods: To address these issues, we introduce an end-to-end framework for automatic sleep stage classification using single-channel EEG: SleepMFormer. At the algorithmic level, SleepMFormer adopts a task-driven simplification of the Transformer encoder to improve attention efficiency while preserving sequence modeling capability. At the training level, supervised contrastive learning is incorporated as an auxiliary strategy to enhance representation robustness. From an engineering perspective, these design choices enable efficient training and inference under resource-constrained settings. Results: When integrated with the SleePyCo backbone, the proposed framework achieves competitive performance on three widely used public datasets: Sleep-EDF, PhysioNet, and SHHS. Notably, SleepMFormer reduces training and inference time by up to 33% compared to conventional self-attention-based models. To further validate the generalizability of MaxFormer, we conduct additional experiments using DeepSleepNet and TinySleepNet as alternative feature extractors. Experimental results demonstrate that MaxFormer consistently maintains performance across different model architectures. Conclusions: Overall, SleepMFormer introduces an efficient and practical framework for automatic sleep staging, demonstrating strong potential for related clinical applications.

## 1. Introduction

Sleep staging serves as a crucial indicator of sleep quality and plays a key part in accurately diagnosing and treating sleep disorders [1]. Currently, polysomnography (PSG) is considered the most reliable method for sleep staging and is widely used for identifying common sleep disorders including narcolepsy, sleep apnea, and sleepwalking [2]. PSG comprises multiple physiological signals, among which EEG, reflecting brain activity, captures the majority of sleep-related features. Sleep specialists manually examine these PSG signals following established sleep scoring rules and classify each 30-s PSG segment (referred to as an “epoch”) into a specific sleep stage. Two frequently applied sleep scoring standards are the Rechtschaffen and Kales (R&K) score rules [3] and the guidelines established by the American Academy of Sleep Medicine (AASM) [4]. The R&K scoring rules classify sleep into Wake (W), Rapid Eye Movement (REM) sleep, and Non-Rapid Eye Movement (NREM) sleep. The NREM stage is further divided into N1, N2, N3, and N4. In contrast, the AASM guidelines combine N3 and N4 into a single N3 stage, defining five distinct sleep stages, which currently serves as the prevailing criterion in sleep staging. Manual annotation of sleep stages is labor- and time-intensive [5], requiring sleep experts nearly two hours to annotate a full night of PSG recordings. In contrast, machine learning algorithms can accomplish sleep staging in a fraction of the time [6,7]. Hence, developing an accurate and efficient automatic sleep staging algorithm is of paramount importance [8].

EEG signals during different sleep stages exhibit complex temporal and spectral patterns, often with considerable similarities [4]. For instance, sleep spindles observed in N2 and N3 stages exhibit frequency ranges and waveform features similar to those of alpha waves present in stage N1. Accurate automatic sleep staging driven by EEG remains challenging due to these subtle differences and the complex dynamics underlying sleep transitions. In addition, EEG-based sleep staging methods often encounter difficulties distinguishing between REM and N1 stages due to the absence of eye movement information, which results in high similarity of them. Moreover, the classification of certain sleep stages does not solely depend on the features of the current epoch, but also relies on contextual information from preceding epochs. Early automatic sleep staging algorithms relied on hand-crafted feature extraction based on expert knowledge, followed by traditional machine learning classifiers [9,10]. However, these methods were typically labor-intensive, required extensive domain expertise, and their performance was largely relied on the quality of the manually designed features.

Recent studies have increasingly adopted deep learning approaches to replace manual feature extraction in sleep staging [11,12]. Early methods primarily relied on convolutional neural networks (CNNs) [6,13] to automatically learn representations from raw EEG signals. A representative work, DeepSleepNet [7], introduced a dual-path CNN to capture sleep patterns at multiple temporal resolutions, inspiring subsequent models [14,15] that explored hierarchical feature extraction. Following the design of U-Net [16], U-Time [17] adopted a sequence-to-sequence architecture aligning more closely with the temporal nature of sleep staging. According to the AASM guidelines [4], contextual information across adjacent epochs is crucial, motivating the adoption of sequence modeling architectures. Early attempts used recurrent networks, particularly LSTMs, to model temporal dependencies in sleep data. TinySleepNet [18] combined CNN-based feature extraction with lightweight RNNs, while SleepEEGNet [19] employed multi-scale CNNs followed by bidirectional RNNs. IITNet [20] further advanced this direction by jointly modeling intra-epoch and inter-epoch dependencies, producing richer temporal representations. Meanwhile, spectrogram-based models such as XSleepNet [21] and SleepTransformer [11] incorporated frequency-domain information to capture stage-specific spectral patterns, but these transformations can cause information loss and increase preprocessing and computational costs.

More recently, contrastive learning has been introduced to improve EEG representation learning. Self-supervised methods [22,23] demonstrated that contrastive objectives can extract meaningful EEG features without explicit labels, while CoSleep [24] extended this concept to multi-view contrastive learning between raw signals and spectrograms. However, these approaches neglect label information, limiting their discriminative capability. Supervised contrastive learning [25] was subsequently proposed to address this limitation, and SleepyCo [12] applied it to enhance intra-epoch representations, though it did not fully leverage inter-epoch or multi-scale dependencies essential for accurate staging. In parallel, attention-based models have gained traction for long-sequence modeling; however, the quadratic computational complexity $O(n^{2})$ of self-attention limits scalability for long EEG sequences. To address this, recent studies such as BigBird [26] and AgentAttention [27] introduced sparse and hybrid attention mechanisms to improve modeling efficiency without compromising representational capacity. These methods are primarily proposed as general-purpose efficient attention mechanisms for long-sequence modeling [28,29,30]. They typically rely on low-rank projections, kernel-based approximations, or predefined sparse attention patterns to reduce computational complexity, which may not be optimal for sleep EEG signals characterized by strong temporal redundancy and stage-dependent amplitude patterns.

To overcome these challenges, we introduce an end-to-end multi-epoch sleep staging algorithm incorporating attention mechanism: SleepMFormer. Rather than proposing a new universal low-complexity attention formulation, SleepMFormer focuses on a task-driven simplification of the Transformer encoder, explicitly tailored to the temporal structure and signal characteristics of sleep EEG. SleepMFormer directly processes raw EEG signals, eliminating the need for complex preprocessing steps and manual feature engineering while generating accurate sleep stage predictions. Specifically, SleepMFormer consists of two parts: the model backbone and the temporal modeling module. For the model backbone, we adopted the existing three model parameters-based hierarchical network from small to large: TinySleepNet [18], DeepSleepNet [7], and SleePyCo [12]. For the temporal modeling module, we reduced the sequence lengths of K and V through max pooling to form an improved Transformer Encoder. In addition, we incorporate supervised contrastive learning [25] at multiple scales and across epochs, simultaneously enhancing intra-epoch and inter-epoch feature representations. To summarize, this work makes the following principal contributions:

- We introduce SleepMFormer, an innovative single-channel EEG framework for sleep staging that achieves state-of-the-art performance across three public datasets: Sleep-EDF, PhysioNet, and SHHS.
- We introduce an efficient attention module tailored for sleep staging, significantly reducing computational overhead while maintaining strong performance.
- We employed a supervised contrastive learning approach to enhance the feature representations of intra-epoch and inter-epoch, thereby improving the classification accuracy.
- We conduct comprehensive ablation analyses and visualization interpretation to verify the contribution of each component and assess various modeling strategies.

The following sections are arranged as follows. Section 2 presents the SleepMFormer framework. The training procedure is described in Section 3. Section 4 details the experimental setup. The results and discussions are detailed in Section 5. Section 6 provides further analysis and interpretation of the model. Finally, Section 7 concludes the paper and outlines future research directions.

## 2. Model Architecture

### 2.1. Problem Formulation

SleepMFormer is developed as an end-to-end framework, processing *L* consecutive single-channel EEG epochs to determine the sleep stage for the *L*th epoch (referred to as the target EEG epoch). We represent a sequence of *L* consecutive EEG epochs, sampled at rate *F*, as $X^{\left(L\right)}\in R^{1\times D\cdot F\cdot L}$, where *D* (seconds) represents the duration of each EEG epoch. The sequence $X^{\left(L\right)}$ is defined as $X^{\left(L\right)}=\left\{x_{1},x_{2},\ldots x_{L}\right\}$, where each $x_{i}\in R^{1\times D\cdot F}$ represents the *i*th EEG epoch.

Following AASM standards [4], we define $N_{c}=5$ sleep stages, corresponding to {Wake, N1, N2, N3, REM}. The predicted sleep stage for the target EEG epoch is represented as $\hat{y}\in {\left\{0,1\right\}}^{N_{c}}$ and $\sum_{j=1}^{N_{c}}\hat{y}=1$. The task of sleep stage prediction can be framed as a multi-class classification problem, aiming to learn a mapping function $f:X^{\left(L\right)}\to \hat{y}$.

### 2.2. Overview

**Figure 1:**
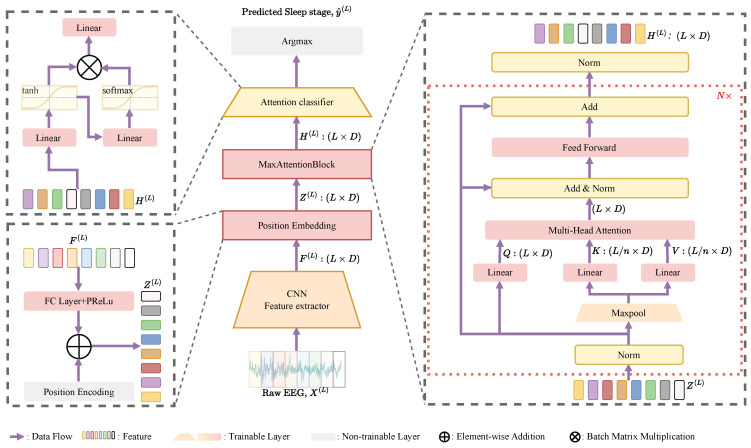
Model Architecture of SleepMFormer.

Figure 1 depicts the SleepMFormer architecture, consisting of three primary components: (1) a Feature Extractor (FE), (2) a Transformer-based Sequence Encoder (TSE), and (3) an Attention-based Sleep Stage Classifier (AS2C). First, the feature extractor (FE) converts the input EEG epochs into feature sequences. Subsequently, these features undergo embedding and positional encoding prior to being fed into the TSE, which captures the sequential relationships among epochs and extracts sequence-level features. Finally, the AS2C module infers the target sleep stage from the encoded sequence of features. The TSE adopts a MaxFormer encoder composed of 6 layers, with an embedding dimension of 128 and 8 attention heads. The architectural details of each module are presented in the subsequent sections.

### 2.3. Feature Extractor

To verify the effectiveness of our proposed framework, we employed three existing feature extraction backbone networks: TinySleepNet [18], DeepSleepNet [7], and SleePyCo [12]. Table 1 presents a comparison of the number of parameters and floating point operations (FLOPs) for these three backbone networks. TinySleepNet is a lightweight backbone network with relatively fewer parameters. In contrast, DeepSleepNet has more model parameters and employs two different-sized convolution kernel functions to achieve the dual-scale observation effect of the model on the data, thereby better adapting to the different frequency sleep features in different sleep stages. Furthermore, SleePyCo is a relatively complex backbone network. Inspired by the feature pyramid network used in object detection [31], SleePyCo integrates a feature pyramid architecture to promote multi-scale feature learning, thereby extracting hierarchical features from the electroencephalogram signals and enhancing the discrimination ability in different sleep stages. Additionally, SleepyCO also integrates the contraction and excitation (SE) block [32] to enhance the model’s representational capability.

Each feature extraction module is followed by a dimension transformation module, which ensures that the features output by different feature extractors have the same dimension to guarantee the consistency of comparison in the subsequent temporal enhancement module. The dimension transformation module also ensures that the different scale features in SleePyCo have the same dimension, so that the same temporal enhancement module and classifier can process the features of different scales. The dimension transformation module is implemented through one-dimensional convolution.

Formally, let $X^{\left(L\right)}$ denote *L* consecutive EEG epochs, the FE architecture is constructed as described below:

(1) $$\begin{array}{c} F^{\left(L\right)}=\mathrm{FE}\left(X^{\left(L\right)}\right) \end{array}$$

### 2.4. Transformer-Based Sequence Encoder

According to AASM guidelines, experts generally evaluate surrounding epochs when determining the sleep stage. To effectively capture the sequential relationships among EEG epochs, we design a Transformer-based Sequence Encoder (TSE) module, as shown in Figure 1, to models these relationships and extracts sequence features. Recent studies have successfully leveraged transformer-based models to encode sequence features from sleep EEG signals [11,12]. Compared to traditional RNN- [33] and LSTM-based [34] models, Transformer-based architectures demonstrate superior performance in capturing long-range dependencies among EEG epochs.

Before entering the TSE module, the feature sequences generated by the FE undergo processing through a common fully connected (FC) layer that utilizes PReLU nonlinearity [35]. This layer projects EEG representations from multiple convolutional scales into a unified embedding space, enabling the shared classifier to accurately model temporal relationships across different feature levels. In particular, we use i=3,4,5 to represent the different sequence lengths of the feature pyramid of SleePyCo. For the other two backbone networks, we only take one value. Therefore, the output from the shared FC layer is represented as ${\tilde{F}}_{i}^{\left(L\right)}\in R^{\left[d_{f}\times 3000L/r_{i}\right]}$, where $d_{f}$ indicating the channel size of the pyramidal features. Subsequently, because the Transformer model requires fixed-length input sequences, positional encoding (PE) is employed to augment the feature sequences to supply the model with information about their positions. As shown in Figure 1, the PE is combined with the input feature vectors, allowing the model to distinguish the order of EEG epochs in a sequence. The encoded feature sequence $Z_{i}^{\left(L\right)}$ corresponding to the *i*-th pyramidal feature representation is defined as:

(2) $$\begin{array}{c} Z_{i}^{\left(L\right)}={\tilde{F}}_{i}^{\left(L\right)}+P_{i}^{\left(L\right)}, \end{array}$$

where $P_{i}^{\left(L\right)}\in R^{\left[d_{f}\times 3000L/r_{i}\right]}$ represents the PE associated with the *i*-th feature vector. Our model employs sinusoidal PE, as adopted in prior research [36]. To accommodate feature sequences of differing lengths sharing the same time indices, we altered the positional encoding by hopping temporal indices so it corresponds to their absolute temporal positions. Therefore, the element of $P_{i}^{\left(L\right)}$ at temporal position *t* in the *j*-th feature sequence is expressed as:

(3) $$\begin{array}{c} P_{i}^{\left(L\right)}\left(t,j\right)=\left\{\begin{array}{cc} \sin\left(\frac{tR^{i-3}+\left\lfloor R^{i-3}/2\right\rfloor }{{10,000}^{2j/d_{f}}}\right), & \mathrm{if}\;j\;\mathrm{is}\;\mathrm{even},\\ \cos\left(\frac{tR^{i-3}+\left\lfloor R^{i-3}/2\right\rfloor }{{10,000}^{2j/d_{f}}}\right), & \mathrm{if}\;j\;\mathrm{is}\;\mathrm{odd}. \end{array}\right. \end{array}$$

where ⌊·⌋ represents the floor function, and $R=r_{i}/r_{i-1}$ (set to 5 in SleePyCo) determines the transition rate between consecutive feature levels *i*-th and (i−1)-th. For i=3, $P_{i}^{\left(L\right)}\left(t,j\right)$ reduces to the original sinusoidal PE.

**Figure 2:**
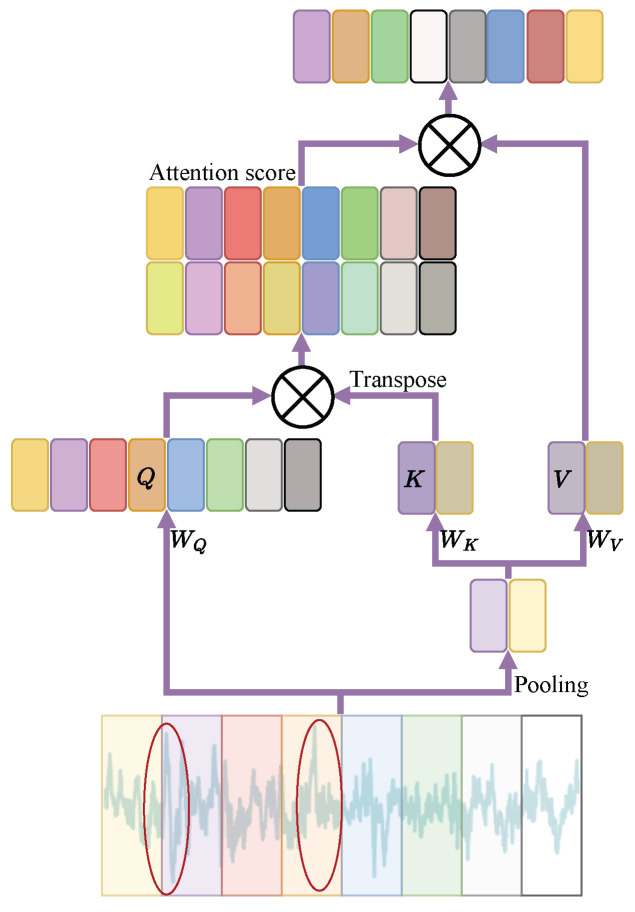
Illustration of the max-attention mechanism.

The TSE module integrates two key components: a multi-head strided self-attention mechanism and a feed-forward neural network (FFNN). The first part aims to model long-range relationships across EEG epochs within a sequence, while the FFNN is used to model the non-linear relationship among EEG epochs. To ensure information completeness, conventional self-attention mechanisms exhibit a quadratic complexity of $O(n^{2})$. This is because, in applications such as image and language modeling, preserving all information is typically crucial. In EEG-based sleep staging, however, the signals frequently exhibit significant redundancy, where recurring patterns across different sleep stages may introduce classification ambiguity at the task level. For instance, both N1 and REM stages exhibit low-amplitude mixed-frequency (LAMF) activity, while sparse alpha activity in N1 and sleep spindles in N2 may further increase confusion between stages. Inspired by the approach in [27], we reduce the sequence length of the keys and values by applying max pooling, and compute temporal attention through broadcasting and querying of the queries. EEG amplitude is an important factor in sleep staging, as different stages exhibit distinct amplitude patterns. From an engineering perspective, max pooling is adopted as a simple reduction operator to emphasize high-response temporal components that are often informative for sleep stage discrimination. As illustrated in Figure 2, the proposed mechanism enables the network to emphasize task-relevant temporal patterns while suppressing redundant information in the context of downstream sleep stage classification.

The strided attention mechanism is defined below:

(4) $$\begin{array}{cc} \mathrm{MultiHead}(Q,K,V) & =\mathrm{Concat}\left({\mathrm{Attention}}_{1},\ldots ,{\mathrm{Attention}}_{h}\right)W^{O},\\ Q & =W_{Q}Z_{i}^{\left(L\right)},\\ A^{\left(L/n\right)} & =\mathrm{MaxPool}(Z_{i}^{\left(L\right)}),\\ K & =W_{K}A^{\left(L/n\right)},\\ V & =W_{V}A^{\left(L/n\right)}, \end{array}$$

where Q,K,V represent to the query, key, and value matrices. $W^{Q}$, $W^{K}$, and $W^{V}$ represent the *i*-th query, key, and value projection weights. The parameter *n* determines both the kernel dimensions and stride length in the max-pooling operation.

(5) $$\begin{array}{c} \mathrm{Attention}\left(Q,K,V\right)=\mathrm{softmax}\left(\frac{QK^{T}}{\sqrt{d_{k}}}\right)V, \end{array}$$

where $d_{k}$ represents the dimensionality of the query, key, and value matrices. For $Z_{i}^{\left(L\right)}\in R^{\left[d_{f}\times 3000L/r_{i}\right]}$, the $A\in R^{\left[d_{f}\times 3000L/r_{i}\times n\right]}$ is the agent matrix, we set l=L/n simply for the sake of simplicity. By preserving the information integrity of *Q* matrices and leveraging *K* matrices and *V* matrices to extract the most salient features, the subsequent attention-based querying and aggregation reduce information loss while enhancing the saliency of critical features. This process decreases the computational cost of the attention mechanism from $O(L^{2}d)$ to O(Lld), thereby significantly improving computational efficiency.

The FFNN module incorporates two FC layers featuring PReLU activation [35], with an additional residual connection. In the TSE module, we choose $d_{f}$ as the output dimension of *Q*, *K*, and *V* matrices in multi-head attention. The dimension ratio between the feedback network and $d_{f}$ is set to 1. In summary, the TSE module can be described as:

(6) $$\begin{array}{cc} H_{i}^{\left(L\right)} & =\mathrm{TSE}(Z_{i}^{\left(L\right)}). \end{array}$$

### 2.5. Attention-Based Sleep Stage Classifier

The Attention-based Sleep Stage Classifier (AS2C) module aims to classify the sleep stage using the encoded feature sequence. The AS2C module consists of an attention mechanism fol lowed by a FC layer. Following the approach in SeqSleepNet [37] and Sleepyco [12], an attention layer [38,39] is used to aggregate the TSE module’s hidden states into a unified feature representation. Initially, the output states $H_{i}^{\left(L\right)}$ are converted to attention states $A_{i}^{\left(L\right)}=\left\{a_{i,1},a_{i,2},\ldots ,a_{i,t_{i}}\right\}$, where $t_{i}=\left\lceil 3000L/r_{i}\right\rceil$, through a single FC layer. Subsequently, the attention states ${\bar{a}}_{i}$ for the *i*-th pyramidal feature sequence is computed by temporally aggregating the attention states through weighted summation.

(7) $$\begin{array}{c} {\bar{a}}_{i}=\sum_{t=1}^{T_{i}}\alpha_{i,t}a_{i,t}, \end{array}$$

where $\alpha_{i,t}$ represents the attention weight corresponding to the *t*-th hidden state within the *i*-th pyramidal sequence. The temporal weight at step *t* is calculated by normalizing attention scores through softmax activation:

(8) $$\begin{array}{c} \alpha_{i,t}=\frac{\exp(e_{i,t})}{\sum_{t=1}^{T_{i}}\exp\left(e_{i,t}\right)}, \end{array}$$

where $e_{i,t}\in R^{1\times d_{f}}$ is the attention score for the *t*-th hidden state in the *i*-th pyramidal sequence.

After computing the attentional feature vector ${\bar{a}}_{i}$, a FC layer produces the output logits for the *i*-th pyramidal sequence:

(9) $$\begin{array}{c} o_{i}=W_{a}{\bar{a}}_{i}+b_{a}, \end{array}$$

where $W_{a}$ represents the FC layer and weight matrix, while $b_{a}$ corresponds its bias vector. Finally, the predicted sleep stage $\hat{y}$ is obtained by integrating the output logits from the three pyramidal sequences:

(10) $$\begin{array}{c} \hat{y}=\mathrm{argmax}\left(\sum_{i=3}^{5}o_{i}\right). \end{array}$$

## 3. Training Procedure

**Figure 3:**
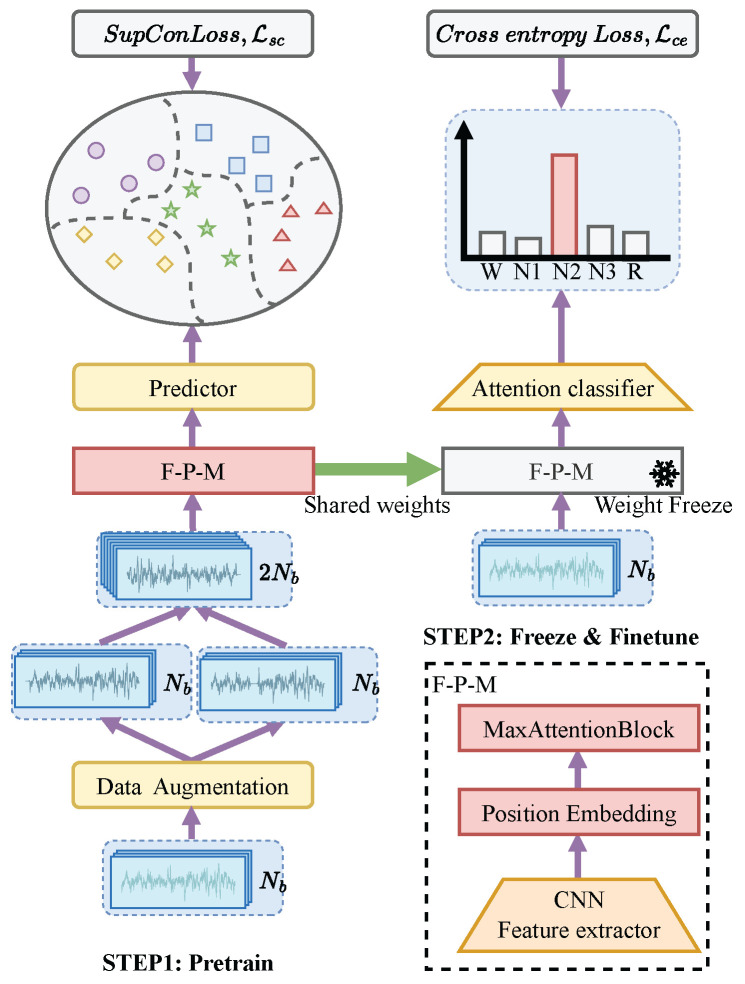
Representation learning via supervised contrastive loss.

Specifically, our training framework consists of two separate phases. As shown in Figure 3, we first train the model except for the AS2C module using the Supervised contrastive (Supcon) representation learning. During this phase, we aim to learn the representation of EEG epochs that captures the sequential relationships among them based on Supcon loss. The second phase is to fine-tune the AS2C module by minimizing cross-entropy loss. Based on empirical observations on training stability and performance consistency, we preserve the weights of the FE and TSE modules trained by the Supcon loss and freeze them during the fine-tuning process. The AS2C module performs sleep stage classification based on the encoded feature sequence.

To avoid overfitting, early stopping was applied during both training phases by monitoring the validation loss. Therefore, validation is conducted periodically to track the validation loss during training, and the training process terminates when the validation loss shows no improvement across multiple consecutive evaluation periods. The model parameters from the iteration with the minimum validation loss are saved and used as the final model. In our learning framework, early stopping enhances the representational capability of the pre-trained model and prevents overfitting during fine-tuning. Note that we used different validation steps for the two training stages and different datasets.

### 3.1. Supervised Contrastive Learning (SCL)

The Supcon loss [25] is employed as an established training objective to improve representation robustness and class separability from raw EEG signals. As depicted in Figure 3, Supcon learning objective aims to increase the correspondence between paired feature representations derived from augmented versions of identical EEG segments. Simultaneously, the method decreases the similarity between feature projections from distinct sleep stages. To achieve this, we first generate augmented EEG epochs by applying random transformations to the original signals. These augmented epochs are then processed through the FE and TSE modules to obtain multi-scale feature representations. Finally, the projection network maps the extracted multi-scale features onto a hypersphere. Further details are provided in the subsequent sections.

#### 3.1.1. Data Augmentation

The original EEG segments undergo data augmentation to produce two distinct modified versions. Given a randomly sampled batch of data ${\left\{X_{p}^{\left(L\right)},y_{p}\right\}}_{p=1,\ldots ,N_{b}}$ (where $N_{b}$ is the batch size), we apply a series of random transformations to each EEG epoch $X_{p}^{\left(L\right)}$, producing two augmented versions, $X_{p,1}^{\left(L\right)}$ and $X_{p,2}^{\left(L\right)}$. This forms a multiview batch [25], as illustrated in Figure 3. We adopt the same augmentation pipeline as in [12] to generate augmented EEG epochs. This pipeline includes six types of transformations: amplitude shift, amplitude scaling, time shift, zero-masking, a band-stop filter, and additive Gaussian noise. Each transformation is applied with a probability of 0.5. Table 2 summarizes the data augmentation workflow.

#### 3.1.2. Training Modules

The training module is composed of three components: the FE module, the TSE module, and an additional projector, with the classifier being excluded from this configuration. First, we extract multi-scale features from the augmented EEG epochs. These feature vectors are subsequently processed by the backbone network to generate representative embeddings. The projector, a key component of the Supcon learning framework, is responsible for determining the similarity between the projected feature vectors. To achieve this, we employ a multi-layer perceptron (MLP) [40], which comprises two FC layers separated by a ReLU function. The MLP has a single hidden layer with a dimension of 128, ensuring the projected feature vectors have the same dimensionality. Prior to entering the MLP, each multi-scale feature is first undergoes an Adaptive Average Pooling layer to decrease spatial dimensionality, and then flattened into a 1D vector.

#### 3.1.3. Loss Function

We employ the Supcon loss [25] as the objective function for contrastive representation learning. This loss function promotes higher similarity among positive pairs and simultaneously drives apart negative pairs to enhance discriminative ability. In this work, samples sharing the same sleep stage label within multi-view batches form positive pairs, whereas those with differing stage assignments constitute negative pairs. The formulation of the Supcon loss is given by:

(11) $$\begin{array}{c} L_{sc}=-\sum_{p=1}^{2N_{b}}\frac{1}{|P_{p}|}\sum_{q\in P_{p}}\log\frac{\exp(\mathrm{sim}\left(z_{p},z_{q}\right)/\tau )}{\sum_{k\in N_{p}}\exp\left(\mathrm{sim}\left(z_{p},z_{k}\right)/\tau \right)}, \end{array}$$

where $z_{p}$ and $z_{q}$ are the projected feature vectors of the EEG epochs $X_{p,1}^{\left(L\right)}$ and $X_{p,2}^{\left(L\right)}$, respectively. Let $P_{p}$ denote the set of positive examples corresponding to the *p*-th EEG epochs, while $N_{p}$ represents all other samples within the batch excluding the *p*-th one. The similarity function sim(·) computes the cosine similarity between pairs of projected feature representations:

(12) $$\begin{array}{c} \mathrm{sim}\left(z_{p},z_{q}\right)=\frac{z_{p}^{T}z_{q}}{{∥z_{p}∥}_{2}{∥z_{q}∥}_{2}}. \end{array}$$

The temperature parameter $\tau \in R^{+}$ is used to control the sharpness of the similarity distribution (τ=0.07 in all experiments).

### 3.2. Fine-Tuning

As illustrated in Figure 3, after loading the parameters learned by the FE and TSE modules through Supcon learning, these two modules are frozen. Subsequently, the AS2C module is optimized by minimizing the cross-entropy loss, which is mathematically expressed as:

(13) $$\begin{array}{c} L_{ce}=-\sum_{i\in p}\sum_{j=1}^{N_{c}}y_{j}^{\left(L\right)}\log\left(\frac{exp(o_{i,j})}{\sum_{k=1}^{N_{c}}exp\left(o_{i,k}\right)}\right), \end{array}$$

where $y_{j}^{\left(L\right)}$ is the one-hot encoded label corresponding to the *j*-th epoch, $o_{i,j}$ is the output logits of the *i*-th pyramidal feature sequence for the *j*-th EEG epoch. To accommodate the multi-scale characteristics of SleePyCo, p is set to 3, 4, 5, whereas for other feature extractors, p defaults to 1. Since all scale features are generated by a shared classifier, the training process incorporates a broader temporal context. Consequently, Equation (13) more effectively captures the temporal relationships across different scales. As a result, the model enables comprehensive intra-epoch and inter-epoch temporal modeling for EEG epoch signals of length *L*.

## 4. Experiments

### 4.1. Datasets and Preprocessing

To verify the effectiveness and robustness of our model, we performed extensive evaluations using three publicly accessible sleep datasets: Sleep-EDF-153, Physio2018, and SHHS. Table 3 presents key information for each dataset, including subject count, EEG channel used, evaluation protocols, and data distribution for each dataset. In this study, following previous research [11,12,21], we defined each 30-s segment as a single sleep epoch. The EEG signals from all datasets (excluding Sleep-EDF-153) were resampled to 100 Hz and processed with a bandpass filter (0.3–35 Hz). For sleep stages labeled according to the R&K rules [3], we combined N3 and N4 stages into a unified N3 stage. Additionally, for all datasets, non-sleep stages such as MOVEMENT and UNKNOWN were excluded.

**Sleep-EDF-153:** The Sleep-EDF-153 dataset [41] (2018 version) contains 197 PSG recordings comprising EEG, chin EMG, EOG, and event annotations. The dataset is partitioned into two distinct subsets: SC and ST. The first subset includes data from 79 healthy participants aged between 25 and 101 years, whereas the ST subset consists of recordings from 22 subjects who received Temazepam to evaluate its sleep-modifying effects. In our study, we used only the SC subset, as recommended by prior research [11,12,21]. Sleep staging was performed following in the R&K scoring rules [3], where each epoch was labeled into a single annotation from the following sleep stages: WAKE, REM, N1, N2, N3, N4, MOVEMENT, UNKNOWN. To minimize data distribution bias due to an excessive WAKE period, we preserved the sleep data spanning 30 min prior to and following the sleep period. For single-channel sleep staging, we used the FPz-Cz EEG channel.

**Physio2018:** The Physio2018 dataset was provided by the Computational Clinical Neurophysiology Laboratory at Massachusetts General Hospital for the 2018 PhysioNet Challenge [42] on sleep-wake detection. Since the dataset does not include a predefined validation set, we utilized the existing training set, which contains data from 994 subjects aged between 18 and 90 years. Sleep stages were labeled based on the AASM scoring guidelines [4]. For single-channel sleep staging, we utilized the C3-A2 EEG channel for analysis.

**SHHS:** The Sleep Heart Health Study (SHHS) dataset [43,44] is a multicenter cohort investigation focused on exploring how sleep apnea affects cardiovascular disease. This dataset includes two separate phases of PSG recordings, referred to as SHHS-1 and SHHS-2. Each PSG recording includes signals from EEG, EOG, EMG, ECG, as well as two-channel respiratory inductance plethysmography, body position sensors, pulse oximetry, light sensors, and airflow sensors. Sleep staging was performed following in the R&K scoring rules [3], where each epoch was labeled into a single annotation from the following sleep stages: WAKE, REM, N1, N2, N3, N4, MOVEMENT, UNKNOWN. In this study, we utilized the SHHS-1 dataset containing data from 5793 subjects. By deleting some data that had a large number of labels other than sleep stages, we ultimately retained 5760 pieces of data and selected the C4-A1 channel for single-channel sleep staging.

### 4.2. Settings

SleepMFormer employed AdamW optimization [45] with hyperparameters set to: learning rate $\eta =1\times 10^{-4}$, $\epsilon =1\times 10^{-8}$, $\beta_{1}=0.9$, and $\beta_{2}=0.999$. For Supcon learning, we used a batch size of 128, while classification training employed 1024 samples per batch. For the SHHS dataset, validation was performed every 400 training iterations during contrastive learning and every 50 iterations during classification training. For the Physio2018 and Sleep-EDF-153 datasets, validation was conducted every 400 and 50 training iterations for contrastive and classification learning. We implemented early stopping by monitoring validation loss and terminating training after 20 consecutive evaluations without improvement. During cross-validation, we selected the model with the minimum validation loss for final testing. All training was conducted using NVIDIA GeForce RTX 3090 GPUs. All experiments were implemented with Python 3.10.14 and PyTorch 2.2.1.

### 4.3. Evaluation Metrics

The evaluation metrics included Accuracy, Macro-F1 score (MF1), Cohen’s Kappa (κ) [46], and class-wise F1 score. Accuracy measures the proportion of correct predictions relative to the total sample count. The prediction outcomes are categorized as: true positives (TP), true negatives (TN), false positives (FP), and false negatives (FN) Accuracy is commonly computed using the following formula:

(14) $$\begin{array}{c} \mathrm{Accuracy}=\frac{TP+TN}{TP+TN+FP+FN}. \end{array}$$

The Macro-F1 score is calculated as the arithmetic mean of the F1 scores across all classes. The F1 score represents the harmonic mean between precision (PR) and recall (RE), mathematically expressed as:

(15) $$\begin{array}{c} F1=\frac{2\cdot \mathrm{PR}\cdot \mathrm{RE}}{\mathrm{PR}+\mathrm{RE}}, \end{array}$$

with precision (PR) and recall (RE) calculated as follows:

(16) $$\begin{array}{cc} \mathrm{PR}=\frac{TP}{TP+FP}, & \mathrm{RE}=\frac{TP}{TP+FN}. \end{array}$$

The Macro-F1 score is computed as:

(17) $$\begin{array}{c} \mathrm{MF}1=\frac{1}{N_{c}}\sum_{i=1}^{N_{c}}{F1}_{i}, \end{array}$$

where ${F1}_{i}$ represents the F1 metric value computed for class *i*. κ is a statistical metric used to evaluate the agreement between raters on categorical data. It is computed by the formula:

(18) $$\begin{array}{c} \kappa =\frac{p_{o}-p_{e}}{1-p_{e}}, \end{array}$$

where $p_{o}$ refers to the proportion of observed agreement between the raters, equivalent to the model’s accuracy, while $p_{e}$ indicates the expected agreement that would occur purely by chance, $p_{e}$ is computed using the formula below:

(19) $$\begin{array}{c} p_{e}=\sum_{i=1}^{N_{c}}\frac{\left(TP_{i}+FP_{i}\right)\left(TP_{i}+FN_{i}\right)}{{\left(TP+TN+FP+FN\right)}^{2}}, \end{array}$$

### 4.4. Compared Approaches

Our experimental assessment benchmarked SleepMFormer against multiple contemporary methods using identical training protocols:

**CNN** [13] is a convolutional neural network that leverages a single convolutional layer to derive features from EEG recordings.

**SleepEEGNet** [19] integrates two distinct kernel CNNs and sequence-to-sequence architectures to capture both spectral and temporal characteristics extracted from the EEG data. It further utilizes a Bi-RNN to model complex temporal dependencies across consecutive sleep epochs.

**U-time** [17] utilizes a FC network architecture inspired by U-Net, enabling efficient processing of sequential inputs of varying lengths to produce sequence labels at a chosen temporal resolution.

**SleepTransformer** [11] leverages the Transformer Encoder architecture to capture both intra-epoch and inter-epoch temporal dependencies in sleep data.

**SeqSleepNet** [37] is a sequence-to-sequence network that captures temporal dependencies within individual epochs using bidirectional recurrent neural networks (Bi-RNNs). It then models the inter-epoch temporal dependencies by applying Bi-RNNs to the generated feature representations.

**TinySleepNet** [18] is a compact neural architecture that combines convolutional layers with a unidirectional RNN, designed with fewer parameters.

**CNN + LSTM** [47] is a composite architecture integrating convolutional and LSTM layers to extract spatial characteristics and model temporal dependencies in EEG signals.

**IITNet** [20] handles single-channel EEG by segmenting it into fixed-interval subepochs, which are fed into a ResNet to generate feature representations. These representations are then passed to a bi-LSTM to capture temporal dependencies.

**XSleepNet** [21] combine spatial and temporal features of EEG signals using a hybrid architecture that integrates CNNs and LSTMs.

**Sleepyco** [12] uses single-scale contrastive learning to enhance feature extraction within each epoch, and then employs multi-scale features through a Transformer Encoder to capture temporal dependencies across epochs.

## 5. Results

### 5.1. Comparison with State-of-the-Art (SOTA) Methods

**Figure 4:**
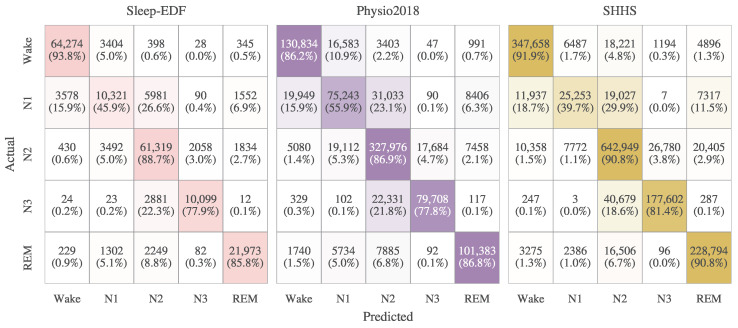
Confusion matrices of SleepMFormer on Sleep-EDF153, Physio2018, and SHHS datasets. The values in parentheses indicate per-class recall and the color intensity indicate the magnitude.

We compared the performance of SleepMFormer with SOTA methods on the three datasets. The results are summarized in Table 4, including number of subjects, the overall metrics (accuracy, MF1, and κ) and the per-class F1 score for each sleep stage. Figure 4 presents the confusion matrices of SleepMFormer for the three datasets. The SleepMFormer model, which uses the SleePyCo feature extractor, produced the top results, performing considerably better than implementations that adopted the DeepSleepNet and TinySleepNet feature extractors on all datasets. Moreover, the DeepSleepNet feature extractor also yielded superior outcomes compared to the TinySleepNet feature extractor. This demonstrates that employing feature extractors with more parameters can enhance model performance.

To be specific, when using SleePyCo as the feature extractor, SleepMFormer achieves competitive performance on the Sleep-EDF153, Physio2018, and SHHS datasets among single-channel EEG–based methods. Quantitatively, SleepMFormer achieved an accuracy of 84.9%, MF1 of 79.3%, and κ of 0.79 on the Sleep-EDF153 dataset, and an accuracy of 81.0%, MF1 of 79.1%, and κ of 0.739 on the Physio2018 dataset. On the SHHS dataset, SleepMFormer achieved an accuracy of 87.8%, MF1 of 80.4%, and κ of 0.826. Compared with strong baseline frameworks, the observed performance differences are relatively small, typically within the range of +0.1–0.3% across accuracy, MF1, and κ on the Sleep-EDF153 and Physio2018 datasets. On the SHHS dataset, SleepMFormer achieves performance levels that are largely comparable to other leading models. When the same framework is employed, using DeepSleepNet for feature extraction produces results nearly identical to those obtained with SleePyCo, and even shows a slight advantage in MF1. While the model utilizing TinySleepNet as the feature extractor continued to exhibit inferior performance relative to the other two extractors, the disparity was notably reduced compared with that observed on the remaining datasets. This suggests that, under identical modeling settings, performance differences among feature extractors diminish as the dataset size increases, with SHHS providing the most stable and saturated evaluation scenario. For more detailed analysis, +1.1 in N1 f1 score and +1.0 in REM f1 score were achieved on the Sleep-EDF153 dataset, +0.5 in N1 f1 score on the Physio2018 dataset and +0.3 in REM f1 score on the SHHS dataset, indicating that SleepMFormer is capable of accurately classifying N1 and REM stages. The framework demonstrates robust cross-dataset performance, enabled by supervised contrastive learning and an efficiency-oriented attention design. By incorporating an attention mechanism that captures both intra-epoch and inter-epoch sequential patterns, coupled with supervised contrastive learning to enhance inter-class feature discriminability, the proposed framework achieves effective feature characterization.

To further assess result stability, we conducted four independent runs using different random seeds. Across the four runs, the overall accuracy, MF1, and Cohen’s κ remain highly consistent under identical experimental settings. On the Sleep-EDF dataset, the macro-F1 score of SleepMFormer-S across repeated runs is 79.28±0.10, with a corresponding Cohen’s κ of 0.790±0.002. On the PhysioNet2018 dataset, the macro-F1 and Cohen’s κ scores are 79.08±0.10 and 0.738±0.002, respectively. Similarly, on the SHHS dataset, SleepMFormer-S achieves a macro-F1 score of 80.33±0.10 and a Cohen’s κ of 0.826±0.003 across four repeated runs. These results indicate stable performance across runs, with only minor variations observed in macro-F1 and Cohen’s κ metrics.

Overall, recent studies have shown that models capturing both intra-epoch and inter-epoch dependencies outperform traditional recurrent architectures such as RNNs and LSTMs, highlighting the importance of long-range temporal modeling in sleep staging. In this context, our framework demonstrates that accurate and efficient sleep staging can be achieved directly from raw EEG signals, without handcrafted features or additional time-frequency transformations, while maintaining stable performance across different backbone extractors. These results suggest that the improvement arises from the model’s intrinsic ability to learn physiologically meaningful temporal patterns rather than from external preprocessing or feature engineering.

### 5.2. Training and Inference Time

**Figure 5:**
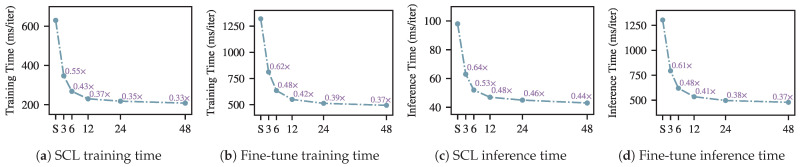
Training and inference time comparison under different pooling strides (*n*). ‘S’ denotes standard self-attention.

The training and inference time of SleepMFormer is shown in Figure 5. All SCL procedures were executed using four NVIDIA GeForce RTX 3090 GPUs with a 128-sample batch configuration. In contrast, the fine-tuning phase was carried out on two RTX 3090 GPUs with a larger batch size of 1024. All training and inference time were measured on the Sleep-EDF153 dataset. As illustrated in Section 2.4, *n* indicates the max-pooling stride of the our attention module and *S* indicates the standard self-attention module.

As shown in Figure 5a,b, With the parameter *n* configured as 6, our framework exhibits significantly improved efficiency, requiring merely 43% for training and 53% for inference of the computational time needed by the self-attention mechanism in SCL. During the fine-tuning phase, the framework requires only 48% of the training and inference time compared to the self-attention mechanism shown in Figure 5c,d. As the stride of max pooling increases, the required processing time decreases. Since the minimum feature sequence length in our multi-scale representation is 48, the maximum pooling stride *n* is set to 48. When n=48, during the contrastive learning stage, the training and fine-tuning time is only 0.51× that of the self-attention mechanism. In the fine-tuning stage, the training and fine-tuning time are only 0.51× and 0.43×, respectively, compared to the self-attention mechanism a substantial improvement. Notably, we also observe that the time reduction becomes limited as the stride continues to increase.

**Figure 6:**
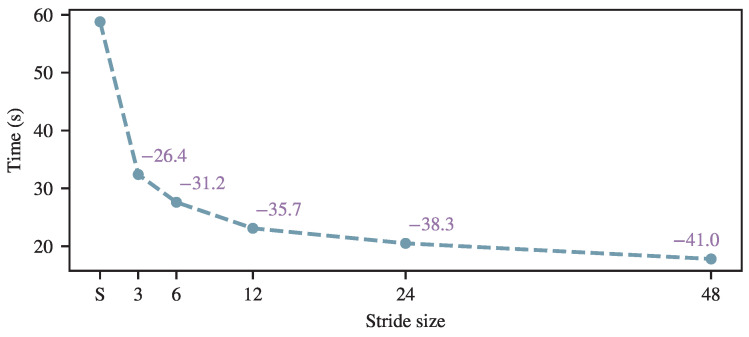
Inference time comparison with CPU under different pooling strides *n* of SleePyCo on the Sleep-EDF dataset. ‘S’ denotes standard self-attention.

To assess inference performance under resource-constrained settings, we additionally report CPU-only results. Experiments are conducted on an Intel Xeon Gold 6226R CPU @ 2.90 GHz, where inference is performed in two successive batches of 512 samples, corresponding to a total of 1024 samples that represent a complete overnight sleep recording. As illustrated in Figure 6, the proposed MaxFormer achieves substantially lower inference latency than the standard Transformer under this configuration. Importantly, this efficiency advantage is further amplified in the CPU-only scenario, as the quadratic self-attention of the standard Transformer incurs a disproportionately higher computational cost, while the max-pooling–based attention design remains comparatively efficient. These results indicate that the efficiency gains are particularly relevant for offline batch sleep scoring on large-scale recordings and for deployment in memory- or computation-constrained environments.

**Figure 7:**
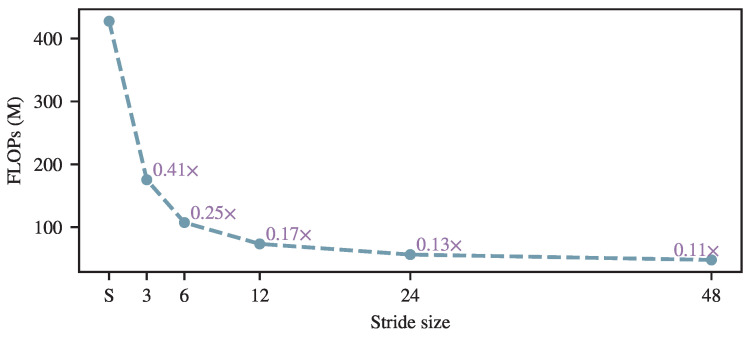
FLOPs comparison under different pooling strides *n*. ‘S’ denotes standard self-attention. All calculations are based on a sequence length of 1200, an embedding dimension of 128, and a batch size of 1.

In addition to the runtime evaluation, Figure 7 reports the FLOPs of the attention module under different pooling strides. This analysis focuses exclusively on the attention operation and shows that the computational complexity decreases monotonically as the stride *n* increases. The observed FLOPs reduction provides a theoretical explanation for the efficiency gains reflected in the training and inference time results. Compared with the runtime measurements, the FLOPs reduction exhibits a more pronounced downward trend, as the analysis isolates the attention module and excludes the influence of the feature extractor and other network components.

### 5.3. Ablation Study

As shown in Table 5, the ablation experiments evaluate two modules: the Attention-based Sleep Stage Classifier (AS2C) and supervised contrastive learning (SCL). In the AS2C module, the conventional fully connected layers are replaced, while in SCL, the model is trained directly without pre-training. Experiments were conducted across three feature extractors—TinySleepNet, DeepSleepNet, and SleePyCo—on all three datasets, using accuracy (ACC), macro F1 score (MF1), and Cohen’s κ as evaluation metrics.

On the Sleep-EDF dataset, the baseline accuracies for the three extractors were 84.1%, 82.0%, and 82.1%, with MF1 scores of 78.1%, 76.4%, and 76.1%, and κ coefficients of 0.779, 0.752, and 0.753, respectively. Applying AS2C led to marginal improvement for TinySleepNet and SleePyCo, while DeepSleepNet gained 0.1% in ACC and 0.8% in MF1, indicating that AS2C helps balance class performance. SCL brought more substantial gains—ACC increased by 0.6%, 1.9%, and 1.4%; MF1 by 1.0%, 1.9%, and 1.7%; and κ by 0.007, 0.025, and 0.019. When both modules were combined, the performance gains were further amplified, showing their complementarity. On the larger PhysioNet dataset, AS2C produced clearer improvements, the ACC was maximally enhanced by 0.3%, and the MF1 was maximally increased by 0.3%. Moreover, by introducing SCL, the ACC and MF1 could be maximally improved by 1.0%, and the minimum improvement could be 0.7% for ACC and 0.5% for MF1. Their combination achieved the best overall results (81.0%, 80.5%, and 80.0% ACC for SleePyCo, DeepSleepNet, and TinySleepNet), confirming that SCL enhances feature discriminability and reinforces attention-based representations. For the SHHS dataset, both AS2C and SCL yielded steady improvements. Baseline accuracies were 87.3%, 87.2%, and 86.4%, with MF1 scores of 80.0%, 80.0%, and 79.0%. Introducing AS2C slightly improved κ (from 0.819 to 0.822), while SCL delivered larger gains—up to 0.4% in ACC and nearly 0.01 in κ. The combination achieved the highest results (87.8%, 87.7%, and 87.2% ACC), demonstrating that contrastive pre-training enhances generalization of attention-based classifiers.

Overall, AS2C mainly strengthens representation focus and class balance, whereas SCL improves inter-class separability and generalization. Together they provide the most stable performance across datasets and extractors, validating their complementary strengths. Notably, SCL exhibits stronger benefits on smaller models and datasets, indicating its effectiveness in mitigating generalization deficiencies caused by limited data and feature capacity.

We further examine the effect of different fine-tuning strategies on model performance using the Sleep-EDF dataset. As shown in Table 6, freezing the feature extractor and Transformer encoder achieves the best overall performance, reaching an accuracy of 84.9%, a macro-F1 score of 79.3, and a Cohen’s κ of 0.790. In contrast, full fine-tuning does not yield consistent improvements and in some cases leads to performance degradation. These results suggest that freezing the encoder provides a stable and effective training strategy for the proposed framework.

### 5.4. Comparison with Standard Transformer Encoder

To further evaluate the robustness of the proposed MaxFormer, we compared its performance with the standard Transformer encoder and a variant using average pooling, as summarized in Table 7. All the experiments were conducted without SCL. MaxFormer achieves comparable performance in most cases across datasets and feature extractors, indicating that the proposed max-pooling–based simplification preserves performance under reduced attention computation.

On the Sleep-EDF dataset, MaxFormer shows small yet consistent improvements in MF1 and κ for some configurations, suggesting better class-balanced behavior under the same evaluation protocol. On the PhysioNet dataset, MaxFormer exhibits competitive performance across different feature extractors. While the most notable improvements are achieved under the DeepSleepNet configuration, the remaining extractors yield results that are largely comparable to those of the standard Transformer, suggesting that MaxFormer maintains robust performance even when improvements are less pronounced. For the large-scale SHHS dataset, MaxFormer consistently preserves the performance of the Transformer encoder and achieves slight yet reliable improvements in accuracy and κ in most configurations, highlighting its robustness on long-duration recordings and large-population cohorts.

In addition, we compare MaxFormer with AvgFormer to examine the impact of different pooling strategies within the attention module. Here, AvgFormer is a variant of MaxFormer in which the max-pooling operation is replaced with average pooling, while all other components remain unchanged. As shown in Table 7, MaxFormer consistently exhibits stronger performance trends than AvgFormer, achieving higher MF1 and κ values in the majority of configurations, while maintaining comparable accuracy. These results indicate that max pooling is more effective than average pooling in preserving discriminative temporal features in EEG sequences, particularly for improving class-balanced performance and agreement metrics.

**Figure 8:**
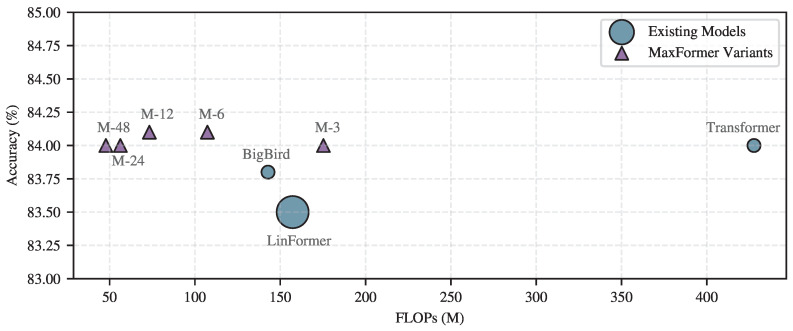
Accuracy versus FLOPs comparison of different efficient attention mechanisms of SleePyCo on the Sleep-EDF dataset. Marker size indicates the number of parameters, and M-*k* denotes a MaxFormer variant with max-pooling stride *k*.

Furthermore, Figure 8 compares MaxFormer with several representative efficient attention mechanisms in terms of classification accuracy and computational cost. The results illustrate the efficiency–performance trade-off achieved by different designs. For MaxFormer, variants with different max-pooling strides are evaluated, where larger strides lead to substantial reductions in FLOPs while maintaining competitive accuracy. For comparison, Linformer employs a fixed low-rank projection with a projection dimension of k=128, while BigBird adopts a sparse attention pattern composed of a local sliding window of size 128, together with 64 random and 16 global attention connections. Notably, MaxFormer variants consistently achieve a favorable balance between accuracy and efficiency, occupying a more advantageous region of the accuracy–FLOPs space compared with other efficient attention approaches.

Overall, these results indicate that MaxFormer can retain, and in most cases enhance, classification performance compared with the standard Transformer encoder, while also demonstrating advantages over the AvgFormer variant. The observed improvements across different datasets and feature extractors suggest that adopting max pooling in the attention module enables more effective modeling of salient temporal dependencies in EEG sequences, particularly in terms of MF1 and κ, while maintaining stable accuracy. Consequently, the proposed sparse-attention design achieves a favorable balance between representational capability and computational efficiency, supporting MaxFormer as a scalable and effective alternative to the conventional Transformer encoder for EEG-based sleep staging.

### 5.5. Effect of Max-Pooling Stride on Model Performance

Max-pooling is inherently an information-losing operation, and this loss becomes more pronounced as the stride increases. Under the extreme condition of n=48, input sequences of original lengths 48, 240, and 1200 are compressed to lengths of 1, 5, and 25, respectively. As shown in Table 8, even under such aggressive downsampling, our model consistently delivers reliable results, as evidenced by its stable accuracy and κ coefficient across evaluations. Notably, across all three datasets, both metrics peak at n=6. Regarding the macro F1 score (MF1), although a slight drop is observed at n=6 on the SHHS dataset, the overall trend remains relatively stable. All three global metrics demonstrate consistent performance across varying stride values. From the class-wise F1 scores, stages W, REM, and N2 appear to be largely unaffected by the stride of max pooling, while N3 and N1 are more sensitive to it. Compared to the standard self-attention mechanism, the N3 stage performance on the Sleep-EDF dataset experiences a marked decline. However, for the N1 stage in Sleep-EDF and both N1 and N3 in the other datasets, the performance remains comparable or even slightly improved. These observations suggest that the features obtained through max pooling with different stride values retain semantically meaningful differences even after upsampling and reconstruction, allowing for accurate classification. Furthermore, we performed max-pooling on the query matrix. Despite applying max pooling with a stride size of 3 to reduce the embedding dimensionality for improved computational efficiency, the model’s performance dropped notably, with accuracy, F1-score, and κ falling to 84.3%, 78.4, and 0.781, respectively. This result highlights the importance of maintaining sufficient embedding capacity in the query representations for effective feature modeling.

**Figure 9:**
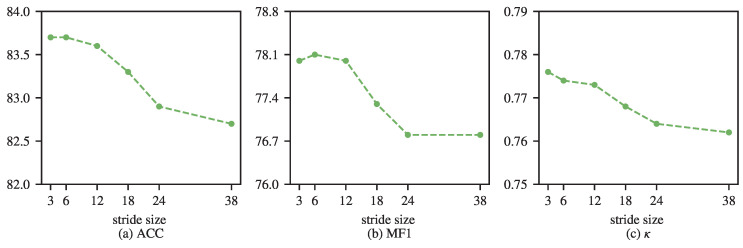
Performance with different pooling strides of TinySleepNet on the Sleep-EDF dataset.

Figure 9 further investigates the influence of pooling stride on performance. For SleePyCo, the performance remains relatively stable when the pooling stride increases, and no abrupt degradation is observed even when the stride reaches 48. This behavior can be attributed to the multi-scale design of SleePyCo, where temporal representations are extracted at three different resolutions. Although the highest-level feature sequence has a length of 48, the remaining two levels preserve longer temporal resolutions of 240 and 1200, which effectively compensate for the information loss introduced by aggressive pooling at the coarsest scale. In contrast, TinySleepNet adopts a single-scale temporal representation. As the pooling stride increases, the effective temporal resolution is progressively reduced, leading to a gradual degradation in classification performance. This trend indicates that large pooling strides can impair temporal modeling when multi-scale representations are not available. These observations suggest that the proposed attention design is more robust when combined with multi-scale feature extractors, while for single-scale backbones, an overly large pooling stride may lead to performance degradation.

### 5.6. Effect of Transformer Encoder Depth

**Figure 10:**
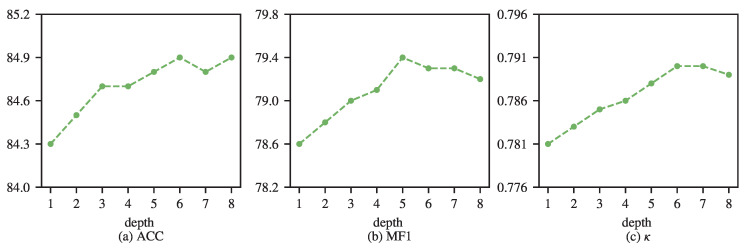
Effect of the number of Transformer encoder layers of SleePyCo on the Sleep-EDF dataset.

As shown in Figure 10, we further examine the impact of the number of Transformer encoder layers on sleep staging performance. Figure 10 summarizes the results obtained with encoder depths ranging from 1 to 8 layers. As the encoder depth increases, classification accuracy shows a gradual upward trend with minor fluctuations, increasing from 84.3% to approximately 84.9%. In contrast, the macro-F1 score improves from 78.6 to a peak value of 79.4 at five layers, after which a slight decline is observed with deeper encoders. A similar trend is observed for Cohen’s κ, which increases from 0.781 to 0.790 at six layers and then marginally decreases as additional layers are introduced. These results indicate that increasing encoder depth improves performance up to a moderate depth, beyond which the benefits saturate and may slightly degrade class-balanced metrics.

## 6. Discussion

### 6.1. Theoretical Analysis of Computational Efficiency

To further elucidate why the proposed MaxFormer significantly accelerates training and inference, we conduct a theoretical analysis of its computational complexity in terms of floating-point operations (FLOPs). The analysis considers both the forward and backward propagation of the attention mechanism and the feed-forward neural network (FFNN).

In a standard Transformer encoder, the dominant computational cost arises from the self-attention module. Given an input sequence of length *L* and feature dimension *d*, the query (*Q*), key (*K*), and value (*V*) projections, together with the output projection, require $4Ld^{2}$ operations. The attention computation includes the matrix multiplication of $QK^{T}$ and the subsequent aggregation with AV, each with a complexity of $O(L^{2}d)$. The FFNN, composed of two linear transformations with an expansion ratio *r*, contributes an additional $2Ld^{2}r$ operations. Consequently, the total FLOPs per Transformer layer in the forward pass can be approximated as:

(20) $${\mathrm{FLOPs}}_{\mathrm{std}}^{\mathrm{fwd}}=\left(4+2r\right)Ld^{2}+2L^{2}d.$$

In the proposed MaxFormer, the key and value matrices are temporally downsampled by a max-pooling stride *n*, reducing their effective sequence length from *L* to L/n. The query matrix remains at full resolution to preserve temporal precision. This design modifies the attention complexity from $O(L^{2}d)$ to $O(L^{2}d/n)$, and the total FLOPs become:

(21) $${\mathrm{FLOPs}}_{\max}^{\mathrm{fwd}}=\left(2+\frac{2}{n}+2r\right)Ld^{2}+2\frac{L^{2}d}{n}.$$

With r=1, n=6, L=1200, and d=128, the theoretical attention cost is reduced by approximately 70% per layer. In addition to the attention computation, the FFNN and normalization layers also benefit from smaller intermediate activations, further reducing the actual wall-clock time during both training and inference.

During training, the backward propagation approximately doubles the computational load of each linear operation because gradients must be computed with respect to both activations and parameters. The total cost per Transformer layer is thus about three times that of the forward pass:

(22) $$\begin{array}{c} {\mathrm{FLOPs}}_{\mathrm{train}}^{\mathrm{std}}\approx 3\times {\mathrm{FLOPs}}_{\mathrm{std}}^{\mathrm{fwd}},{\mathrm{FLOPs}}_{\mathrm{train}}^{\max}\approx 3\times {\mathrm{FLOPs}}_{\max}^{\mathrm{fwd}}. \end{array}$$

Since the gradients of *K* and *V* are computed on the reduced sequence length L/n, the backward cost is proportionally decreased, providing additional efficiency gains during training.

Overall, considering that L≫d in sleep EEG sequences, the quadratic term dominates the total computation, and the expression can therefore be simplified as:

(23) $$\begin{array}{ccc} {\mathrm{FLOPs}}_{\mathrm{std}}^{\mathrm{fwd}} & \approx 2L^{2}d,{\mathrm{FLOPs}}_{\max}^{\mathrm{fwd}} & \approx 2\frac{L^{2}d}{n}. \end{array}$$

This shows that the theoretical complexity of MaxFormer is reduced from $O(L^{2}d)$ to $O(L^{2}d/n)$ while maintaining identical feature dimensionality. The resulting linearized attention effectively suppresses redundant temporal correlations among adjacent EEG epochs and focuses on salient contextual dependencies. This design not only reduces theoretical FLOPs but also achieves practical acceleration in both convergence and inference latency without sacrificing classification accuracy.

### 6.2. Visualization of Attention Weights

**Figure 11:**
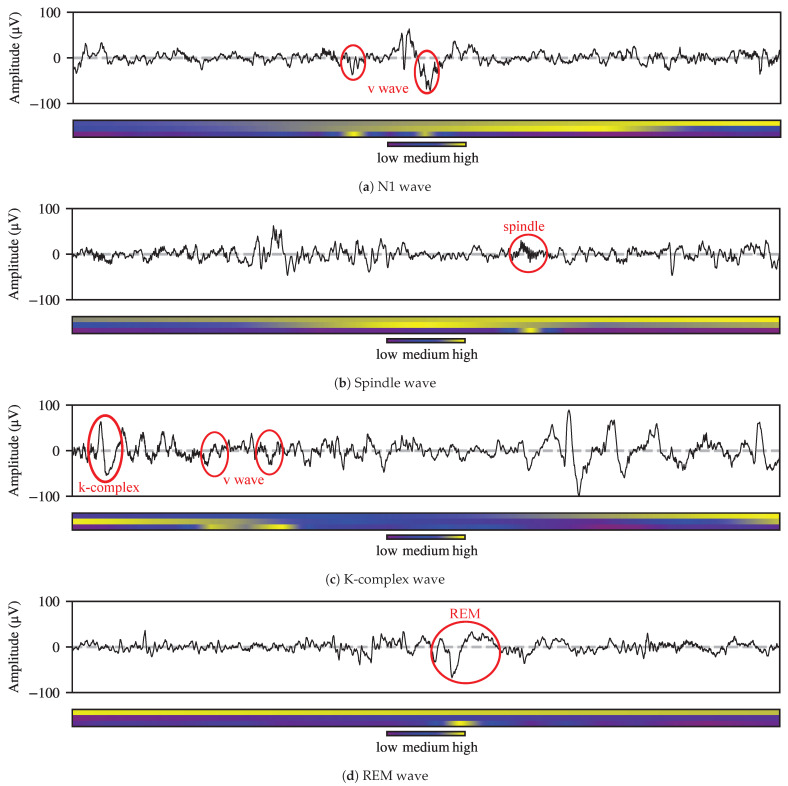
Visualization of attention weights across different sleep stages.

To further verify the effectiveness of the proposed attention mechanism, we take the encoder of SleePyCo as an example and visualize the summed multi-head attention scores of the model configured with n=6. Figure 11 shows four representative examples from the Sleep-EDF validation set. The attention maps are arranged vertically, corresponding to the outputs of different feature scales in the SleePyCo encoder, and are derived from the final layer of the Transformer-based sequence encoder. This visualization aims to demonstrate that our MaxFormer maintains consistent performance across multi-scale representations without losing essential information.

As illustrated in Figure 11a, features at different scales exhibit distinct representational characteristics: deeper scales focus on discriminative waveform patterns (e.g., vertex sharp waves), whereas shallower scales preserve global contextual dependencies. In Figure 11b, the deep features accurately locate sleep spindles during the N2 stage, confirming their sensitivity to stage-specific oscillatory events. In Figure 11c, the signal begins with a micro-arousal followed by a K-complex; the deeper features respond strongly to frequency transitions and sharp waves, the intermediate scale captures the overall arousal process, and the shallow scale reflects the subsequent amplitude variations. Similarly, Figure 11d shows that the deep features effectively capture motion-related artifacts caused by rapid eye movements, which are critical for recognizing REM stages using single-channel EEG.

Overall, these results indicate that the proposed attention mechanism enables the model to preserve complementary information across scales—where deeper layers emphasize fine-grained local events and shallower layers encode the global temporal structure—thus ensuring that multi-scale fusion does not omit key physiological cues in sleep staging.

### 6.3. Clinical Implications and Limitations

Accurate and efficient sleep stage classification is an important component of precision and personalized sleep medicine. By achieving reliable sleep stage discrimination with reduced computational requirements, SleepMFormer is well suited for deployment in low-power and edge-computing scenarios, enabling sleep analysis after data acquisition without reliance on high-performance computing resources. This efficiency makes the proposed framework particularly suitable for home-based sleep monitoring applications, where computational resources are limited and fast model inference is required. In addition, the favorable training efficiency of SleepMFormer facilitates large-scale training on extensive sleep datasets, which is essential for learning robust representations from diverse populations. Nevertheless, the current study focuses on EEG-based sleep staging under standardized experimental settings, and further validation on real-world clinical data is required to fully assess its practical applicability.

## 7. Conclusions

This work presents SleepMFormer, an efficiency-oriented Transformer-based framework for single-channel EEG sleep staging. The contributions of this study can be understood from three complementary aspects: a task-driven simplification of the Transformer encoder for improved efficiency (algorithmic aspect), the adoption of supervised contrastive learning to enhance representation quality (training aspect), and an implementation-oriented integration that facilitates efficient deployment under limited resources (engineering aspect). thereby improving the model’s efficiency and enabling its practical deployment in lightweight or embedded sleep monitoring systems. In addition, the integration of supervised contrastive learning effectively enhances the discriminative power of the learned representations, offering a meaningful approach for representation learning and pretraining in biological time-series analysis. We evaluated SleepMFormer on three widely used public datasets—Sleep-EDF-153, PhysioNet2018, and SHHS—and the results demonstrate that our model achieves comparable accuracy to existing methods while requiring substantially fewer computations. Notably, SleepMFormer achieves competitive performance on the Sleep-EDF-153 and PhysioNet2018 benchmarks while requiring substantially fewer computations. Overall, this work combines an efficiency-oriented attention design with a supervised contrastive training strategy, and emphasizes practical integration with existing sleep staging backbones to enable computationally efficient deployment. Meanwhile, these results highlight the effectiveness of combining efficiency-oriented architectural design with established training strategies, emphasizing practical efficiency–performance trade-offs within the established Transformer framework. In future work, we plan to further streamline the model structure to enhance efficiency and generalizability, including evaluation under cross-dataset and other more challenging data settings, with the long-term goal of achieving robust, real-time, and locally deployable automatic sleep staging systems.

## Figures and Tables

**Table 1:** Comparison of parameters and FLOPs.

Model	Parameters (M)	FLOPs (M)
TinySleepNet [18]	0.41	6.84
DeepSleepNet [7]	0.78	30.60
SleePyCo [12]	1.63	140.06

The FLOPs are calculated based on processing a single 30-s EEG epoch sampled at 100 Hz.

**Table 2:** Data augmentation pipeline.

Transformation	Min	Max	Probability
amplitude shift (μV)	−10	10	0.5 each
amplitude scaling	0.5	2	
time shift (samples)	−300	300	
zero-masking (samples)	0	300	
band-stop filter (2 Hz width) (lower bound frequency, Hz)	0.5	30.0	
additive Gaussian noise (σ)	0	0.2	

**Table 3:** Experimental setup and dataset details.

Dataset	Subjects	Channel	Experimental Setup		Class Distribution					
			Evaluation Scheme	Held-Out Validation Set	W	N1	N2	N3	REM	Total
Sleep-EDF	78	Fpz-Cz	10-fold CV	7 subjects	69,824 (35.0%)	21,522 (10.8%)	69,132 (34.7%)	13,039 (6.5%)	25,835 (13.0%)	199,352
Physio2018	994	C3-A2	5-fold CV	50 subjects	157,993 (17.7%)	136,984 (15.4%)	377,821 (42.3%)	102,592 (11.5%)	116,864 (13.1%)	892,254
SHHS	5960	C4-A1	Train/Test: 0.7:0.3	100 subjects	1,308,982 (24.0%)	246,195 (4.0%)	2,383,133 (43.7%)	735,082 (13.5%)	812,880 (14.9%)	5,456,272

**Table 4:** Performance comparison with existing methods.

Method			Overall Metrics			Per-Class F1 Score				
Dataset	System	Subjects	Acc	MF1	κ	W	N1	N2	N3	REM
Sleep-EDF	SleepEEGNet [19]	78	80.0	73.6	0.73	91.7	44.1	82.5	73.5	76.1
Sleep-EDF	U-Time [17]	78	81.3	76.3	0.745	92.0	51.0	83.5	74.6	80.2
Sleep-EDF	SleepTransformer [11]	78	81.4	74.3	0.743	91.7	40.4	84.3	77.9	77.2
Sleep-EDF	SeqSleepNet [37]	78	82.6	76.4	0.76	-	-	-	-	-
Sleep-EDF	TinySleepNet [18]	78	83.1	78.1	0.77	92.8	51.0	85.3	**81.1**	80.3
Sleep-EDF	CNN + LSTM [47]	78	83.7	-	0.77	-	-	-	-	-
Sleep-EDF	XSleepNet [21]	78	84.0	77.9	0.778	93.3	49.9	86.0	78.7	81.8
Sleep-EDF	SleePyCo [12]	78	84.6	79.0	0.787	93.5	50.4	**86.5**	80.5	84.2
Sleep-EDF	SleepMFormer-T (Ours)	78	83.7	78.1	0.774	93.0	49.2	86.0	80.7	81.4
Sleep-EDF	SleepMFormer-D (Ours)	78	84.0	78.5	0.778	93.0	49.5	86.0	80.6	83.3
Sleep-EDF	SleepMFormer-S (Ours)	78	**84.9**	**79.3**	**0.79**	**93.8**	**51.5**	86.4	79.5	**85.2**
Physio2018	U-Time [17]	994	78.8	77.4	0.714	82.5	59.0	83.1	79.0	83.5
Physio2018	SeqSleepNet [37]	994	79.4	77.6	0.719	-	-	-	-	-
Physio2018	XSleepNet [21]	994	80.3	78.6	0.732	-	-	-	-	-
Physio2018	SleePyCo [12]	994	80.9	78.9	0.737	84.2	59.3	**85.3**	79.4	**86.3**
Physio2018	SleepMFormer-T (Ours)	994	80.0	77.8	0.725	83.2	57.5	84.8	79.8	83.6
Physio2018	SleepMFormer-D (Ours)	994	80.5	78.5	0.732	83.7	58.6	85.0	**80.1**	85.1
Physio2018	SleepMFormer-S (Ours)	994	**81.0**	**79.1**	**0.739**	**84.5**	**59.8**	85.2	79.6	86.2
SHHS	SeqSleepNet [37]	5791	86.5	78.5	0.81	-	-	-	-	-
SHHS	IITNet [20]	5791	86.7	79.8	0.812	90.1	48.1	88.4	**85.2**	87.2
SHHS	CNN [13]	5728	86.8	78.5	0.815	91.4	42.7	88.0	84.9	85.4
SHHS	XSleepNet [21]	5791	87.6	**80.7**	0.826	92.0	**49.9**	88.3	85.0	88.2
SHHS	SleepTransformer [11]	5791	87.7	80.1	0.828	92.2	46.1	88.3	**85.2**	88.6
SHHS	SleePyCo [12]	5760	87.6	80.5	0.823	**92.6**	49.2	88.5	84.5	88.6
SHHS	SleepMFormer-T (Ours)	5760	87.2	79.7	0.818	92.5	48.9	88.7	83.2	**88.9**
SHHS	SleepMFormer-D (Ours)	5760	87.7	**80.7**	0.825	92.2	49.3	88.9	84.5	88.5
SHHS	SleepMFormer-S (Ours)	5760	**87.8**	80.4	**0.826**	92.5	47.9	**89.0**	83.8	**88.9**

‘-’ indicates the corresponding value is not provided. **Bold** indicates the best result. The reported results uniformly utilize our attention architecture with a max-pooling stride of 6 (see Section 2.4). T, D, S indicate TinySleepNet, DeepSleepNet, and SleepMFormer, respectively.

**Table 5:** Ablation study.

Dataset	Method		SleePyCo			DeepSleepNet			TinySleepNet		
	AS2C	SCL	Acc	MF1	κ	Acc	MF1	κ	Acc	MF1	κ
Sleep-EDF	–	–	84.1	78.1	0.779	82.0	76.4	0.752	82.1	76.1	0.753
	✓	–	84.1	78.1	0.780	82.1	77.2	0.754	82.1	76.1	0.754
	–	✓	84.7	79.1	0.786	83.9	78.3	0.777	83.6	77.8	0.772
	✓	✓	84.9	79.3	0.790	84.0	78.5	0.778	83.7	78.1	0.774
PhysioNet	–	–	80.0	78.0	0.725	79.7	77.9	0.723	78.8	76.8	0.700
	✓	–	80.2	78.3	0.728	79.7	77.8	0.723	79.0	76.8	0.713
	–	✓	80.7	78.8	0.735	80.4	78.4	0.731	79.7	77.7	0.722
	✓	✓	81.0	79.1	0.739	80.5	78.5	0.732	80.0	77.8	0.725
SHHS	–	–	87.3	80.0	0.819	87.2	80.0	0.819	86.4	79.0	0.807
	✓	–	87.5	80.0	0.822	87.2	80.3	0.818	86.6	79.1	0.809
	–	✓	87.6	80.4	0.820	87.6	80.6	0.824	87.0	79.9	0.816
	✓	✓	87.8	80.7	0.830	87.7	80.7	0.825	87.2	79.7	0.818

The reported results uniformly utilize our attention architecture with a max-pooling stride of 6 (see Section 2.4).

**Table 6:** Performance comparison under different fine-tuning strategies on the Sleep-EDF dataset.

Dataset	Frozen	SleePyCo			DeepSleepNet			TinySleepNet		
		Acc	MF1	κ	Acc	MF1	κ	Acc	MF1	κ
Sleep-EDF	–	84.6	78.5	0.783	83.4	77.9	0.762	83.4	77.8	0.769
	✓	84.9	79.3	0.790	84.0	78.5	0.778	83.7	78.1	0.774

The reported results uniformly utilize our attention architecture with a max-pooling stride of 6 (see Section 2.4).

**Table 7:** Comparison with standard Transformer encoder.

Dataset	FE	Transformer			AvgFormer			MaxFormer		
		Acc	MF1	κ	Acc	MF1	κ	Acc	MF1	κ
Sleep-EDF	S	84.0	78.0	0.778	83.8 ↓	77.9 ↓	0.776 ↓	**84.1**↑	**78.1**↑	**0.780**↑
	D	81.6	76.3	0.749	81.8 ↑	76.7 ↑	0.750 ↑	**82.1**↑	**77.2**↑	**0.754**↑
	T	81.6	75.9	0.747	81.4 ↓	75.2 ↓	0.743 ↓	**82.1**↑	**76.1**↑	**0.754**↑
PhysioNet	S	**80.2**	**78.3**	**0.728**	80.1 ↓	78.1 ↓	0.725 ↓	**80.2** –	**78.3** –	**0.728** –
	D	79.4	77.6	0.718	79.5 ↑	77.3 ↓	0.719 ↑	**79.7**↑	**77.8**↑	**0.723**↑
	T	**79.2**	**77.2**	**0.716**	78.8 ↓	76.9 ↓	0.712 ↓	79.0 ↓	76.8 ↓	0.713 ↓
SHHS	S	87.4	**80.3**	0.821	**87.5**↑	80.1 ↓	0.820 ↓	**87.5**↑	80.0 ↓	**0.822**↑
	D	87.1	80.2	0.816	**87.2**↑	79.9 ↓	0.818 ↑	**87.2**↑	**80.3**↑	**0.818**↑
	T	86.5	**79.6**	**0.809**	**86.6**↑	79.1 ↓	0.810 ↑	**86.6** ↑	79.1 ↓	**0.809** –

↑, ↓, and – indicate an increase, a decrease, and no change relative to the standard Transformer, respectively. **Bold** values indicate the best result for each metric within the same row. All results employ the proposed attention architecture with a max-pooling stride of 6 (see Section 2.4).

**Table 8:** Performance with different pooling strides of SleePyCo on the Sleep-EDF dataset.

Datasets	*n*	Overall Metrics			Per-Class F1 Score				
		Acc	MF1	κ	W	N1	N2	N3	R
Sleep-EDF	S	84.8	79.3	**0.790**	93.7	51.1	**86.5**	**79.9**	**85.4**
	3	84.8	79.3	0.789	93.7	51.4	**86.5**	79.6	85.1
	6	**84.9**	79.3	**0.790**	**93.8**	51.5	86.4	79.5	85.2
	12	84.8	**79.4**	**0.790**	**93.8**	**51.8**	**86.5**	79.7	85.3
	24	84.8	79.3	0.789	**93.8**	51.6	**86.5**	79.6	84.8
	48	84.8	79.1	0.789	93.7	51.2	**86.5**	79.1	85.1
Physio2018	S	80.9	78.9	0.737	84.2	59.3	**85.2**	79.5	86.0
	3	80.9	78.9	0.738	84.3	59.4	**85.2**	**79.7**	86.0
	6	**81.0**	**79.1**	**0.739**	**84.5**	**59.8**	**85.2**	79.6	86.2
	12	**81.0**	**79.1**	**0.739**	84.3	**59.8**	**85.2**	79.6	**86.3**
	24	80.9	79.0	0.738	**84.5**	59.6	**85.2**	79.6	**86.3**
	48	80.9	78.9	0.738	84.2	59.4	**85.2**	**79.7**	86.1
SHHS	S	87.6	80.5	0.823	92.5	48.9	88.7	83.2	88.9
	3	87.7	**80.7**	0.825	92.5	**49.6**	88.9	83.3	**89.0**
	6	**87.8**	80.4	**0.826**	92.5	47.9	**89.0**	**83.8**	88.9
	12	87.7	80.6	0.825	**92.6**	49.3	88.9	83.3	**89.0**
	24	87.7	**80.7**	**0.826**	92.5	**49.6**	**89.0**	83.7	88.9
	48	87.6	80.5	0.823	92.5	49.5	88.8	82.9	88.9

**Bold** indicates the best result. ‘S’ indicates the standard self-attention module. ‘*n*’ indicates the max-pooling stride of the attention module.

## Data Availability

All datasets employed in this work are publicly accessible through the corresponding repositories. Sleep-EDF is available at PhysioNet: https://physionet.org/content/sleep-edfx/. SHHS can be accessed at the NSRR Sleep Data Repository: https://sleepdata.org/datasets/shhs. Physio2018 is available at PhysioNet: https://physionet.org/content/challenge-2018/. Any additional experimental data or materials generated during the study are available from the corresponding authors upon reasonable request.

## Abbreviations

The following abbreviations are used in this manuscript:

EEG	Electroencephalography
PSG	Polysomnography
AASM	American Academy of Sleep Medicine
R&K	Rechtschaffen and Kales
REM	Rapid Eye Movement
NREM	Non-Rapid Eye Movement
CNN	Convolutional Neural Network
FE	Feature Extractor
TSE	Transformer-based Sequence Encoder
AS2C	Attention-based Sleep Stage Classifier
SCL	Supervised Contrastive Learning
SupCon	Supervised Contrastive Loss
FFNN	Feed-Forward Neural Network
PE	Positional Encoding
FLOPs	Floating-Point Operations
MF1	Macro F1 Score
ACC	Accuracy
CV	Cross-Validation
MLP	Multi-Layer Perceptron
SE	Squeeze-and-Excitation
SHHS	Sleep Heart Health Study
